# [^177^Lu]Lu-DOTA-TATE and [^131^I]MIBG Phenotypic Imaging-Based Therapy in Metastatic/Inoperable Pheochromocytomas and Paragangliomas: Comparative Results in a Single Center

**DOI:** 10.3389/fendo.2022.778322

**Published:** 2022-02-07

**Authors:** Stefan Prado-Wohlwend, María Isabel del Olmo-García, Pilar Bello-Arques, Juan Francisco Merino-Torres

**Affiliations:** ^1^ Nuclear Medicine Department, University and Polytechnic Hospital La Fe, Valencia, Spain; ^2^ Endocrinology and Nutrition Department, University and Polytechnic Hospital La Fe, Valencia, Spain; ^3^ Medicine Department, Universitat de València, Valencia, Spain

**Keywords:** paraganglioma, pheochromocytoma, [^131^I]MIBG, PRRT, [^68^Ga]Ga-DOTA-TOC, phenotypic imaging, [^177^Lu]Lu-DOTA-TATE

## Abstract

**Purpose:**

The aim of the study is to assess phenotypic imaging patterns and the response to treatment with [^177^Lu]Lu-DOTA-TATE and/or [^131^I]MIBG in paragangliomas (PGLs) and pheochromocytomas (PHEOs), globally and according to the primary location.

**Methods:**

This is a 17-patient retrospective observational study, with 9 cases treated with [^177^Lu]Lu-DOTA-TATE and 8 with [^131^I]MIBG (37 total treatments). Functional imaging scans and treatment responses were studied in order to choose the best therapeutic option and to define the progression-free survival (PFS) and disease control rate (DCR) according to treatment modality and primary location.

**Results:**

All patients were studied with phenotypic nuclear medicine images. Twelve of 17 patients were tested with both [^123^I]MIBG and somatostatin receptor images, and 6/12 showed appropriate expression of both targets to treatment in the phenotypic images. The rest of the patients were tested with one of the image modalities or only showed suitable uptake of a single radiotracer and were treated with the corresponding therapeutic option. [^177^Lu]Lu-DOTA-TATE PFS was 29 months with a DCR of 88.8%. [^131^I]MIBG PFS was 18.5 months with a 62.5% DCR. According to the primary location, the best PFS was in PHEOs treated with [^177^Lu]Lu-DOTA-TATE. Although the series are small due to the low disease prevalence and do not allow to yield statistically significant differences, this first study comparing [^177^Lu]Lu-DOTA-TATE and [^131^I]MIBG displays a trend to an overall longer PFS with [^177^Lu]Lu-DOTA-TATE, especially in the adrenal primary location. When both radionuclide targets are expressed, the patients’ comorbidity and treatment effectiveness should be valued together with the intensity uptake in the phenotypic image in order to choose the best therapeutic option. These preliminary retrospective results reinforce the need for a prospective, multicentric trial to be confirmed.

## Introduction

Paragangliomas (PGLs) and pheochromocytomas (PHEOs) collectively abbreviated as PGGLs are infrequent neuroendocrine tumors derived from chromaffin cells of the adrenal medulla and extra-adrenal sympathetic or parasympathetic ganglia that can be located from the skull to the sacrum.

Within this group, the term PHEO is reserved for a PGL derived from the adrenal medulla. Of PGGLs, 80%–85% are PHEOs, and approximately 15%–20% are PGLs (1). Most PGGLs related to the sympathetic nervous system secrete catecholamines (85%) in contrast to most PGGLs derived from the parasympathetic, which are mostly non-functioning.

The yearly incidence of overall PGGLs is between 1 and 2 cases per million, out of which 5%–20% of PHEOs and 15%–35% of PGLs present an advanced stage of the disease. Approximately 5%–10% are solitary, and the presence of metachronous or synchronous extra-adrenal PGLs is associated with germline or somatic mutations. PGGLs have a significative hereditary predisposition, with 30%–40% of them determined by a germinal autosomal dominant mutation. Depending on the mutation, they will show a specific phenotypic pattern in the images (2). So far, 32 genes related to this disease have been identified. SDHx are the most frequent mutations among metastatic PGGLs in adults (43%–71%) and children (70%–82%), followed by VHL mutations (1%–13%), whereas the cumulative frequency of RET, NF1, TMEM_127_, and MAX is reported to be between 1% and 11% (3).

In patients with advanced disease, the goal of therapy is to reduce the tumor burden and to control the symptoms. In this context, the nuclear medicine theranostic radiopharmaceuticals play an important role in personalized health strategies, tailoring diagnosis and treatment for each patient and creating an emerging clinical dilemma to select the most suitable targeted radionuclide therapy (4, 5).

In order to solve this question, nuclear medicine phenotypic images are able to reveal tumor characteristics at the molecular and genetic levels reflecting the expression of certain target receptors or transporters (6).

**Figure 1:**
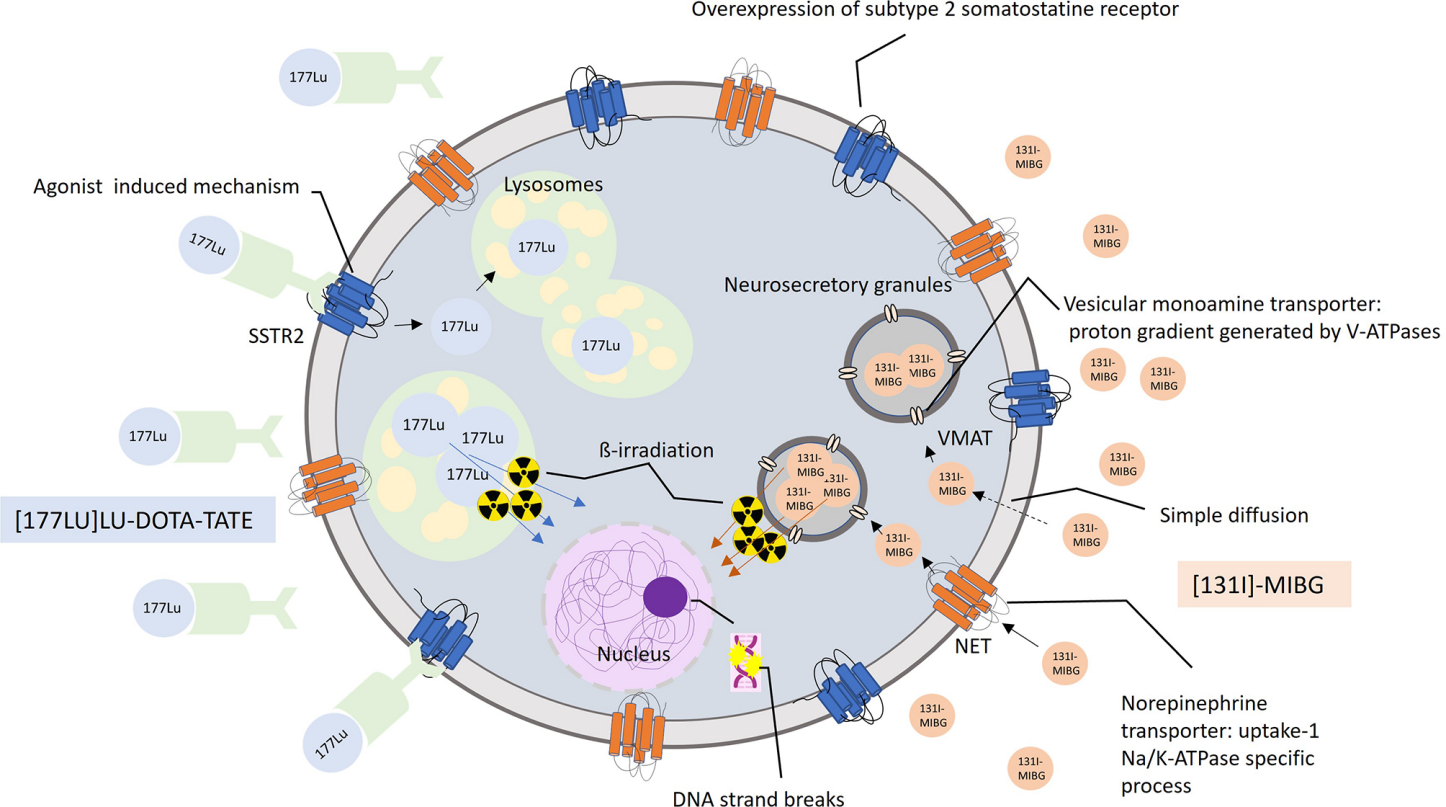
Mechanism of action in [^177^Lu]Lu-DOTA-TATE and [^131^I]-MIBG radiopharmaceuticals.

The two main systemic available radiolabeled therapies in PGGLs are [^177^Lu]Lu-DOTA-TATE, also called peptide receptor radionuclide therapy (PRRT), which binds to somatostatin receptors (SSTRs), and [^131^I]MIBG, which targets the norepinephrine transporter system (NET) (**Figure 1**).

[^177^Lu]Lu-DOTA-TATE contains a semisynthetic somatostatin analog (SSA) with high affinity particularly for SSTR subtype 2 (SSTR2), bound through to a beta emitter ^177^Lu isotope. This radiopharmaceutical binds to SSTR2s, which are overexpressed in at least 80% of the neuroendocrine tumors, enters within tumor cells, and is stored in the lysosomes, thereby causing cellular death by breaking DNA strands.

[^131^I]MIBG (low-specific-activity (LSA) MIBG) is a norepinephrine analog and adrenergic neuron blocker agent that enters neuroendocrine cells in two ways: a specific NET-mediated one with a high affinity and a passive diffusion mechanism with low affinity. Inside the cell, [^131^I]MIBG is stored *via* vesicular monoamine transporter (VMAT) in neuroendocrine vesicles emitting beta particles.

**Figure 2:**
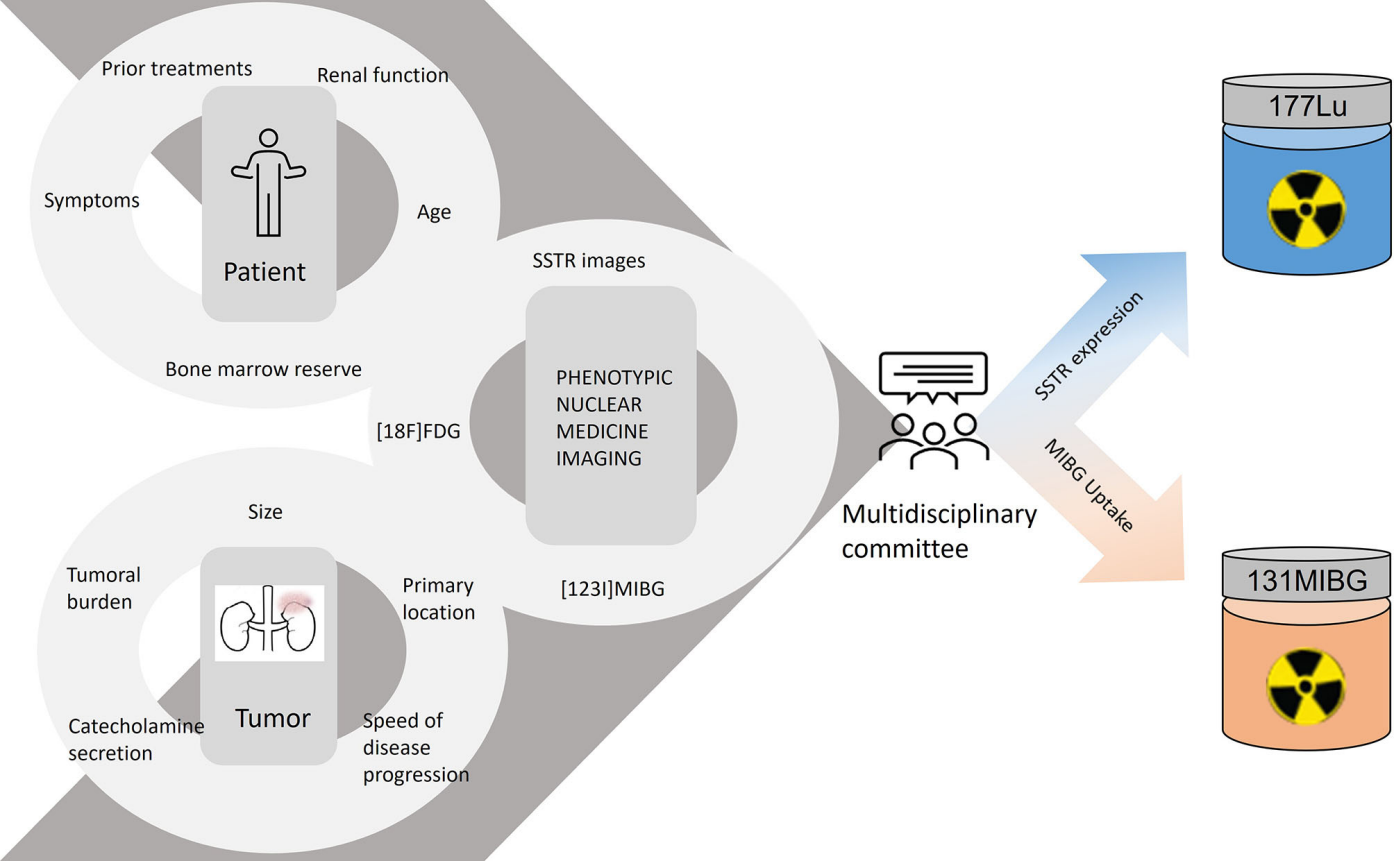
Therapeutic decision workflow.

To select whether to administer one or another radiolabeled treatment, the phenotypic nuclear medicine images signal the expression of the therapeutic targets. A patient can display appropriate uptake of one or both radiotracers, and in the latter scenario, the uptake can show a heterogeneous pattern, increasing the complexity of the therapeutic decision. In addition to the phenotypic images, we must also assess the patient’s profile (age, previous treatment, and bone marrow reserve) and the characteristics of the tumor (volume, location, hormonal secretion, and growth rate), so as to choose the best treatment option (**Figure 2**).

## Material and Methods

The primary aim of this report is to study the response to treatment in terms of progression-free survival (PFS) and disease control (DC) rate (DCR), with [^177^Lu]Lu-DOTA-TATE and/or [^131^I]MIBG in PGLs and PHEOs, globally and according to the primary location. The secondary aim is to assess the phenotypic imaging-based selection to treatment and to evaluate which patients were candidates for both treatments.

This is a retrospective observational study involving 17 patients with histologically proven metastatic and/or inoperable PHEOs and PGLs, referred to our hospital for radionuclide therapy from April 2014 until May 2021. Nine patients were treated with PRRT and 8 with [^131^I]MIBG. A total of 37 treatment cycles were performed (28 PRRT and 9 [^131^I]-MIBG). A multidisciplinary committee evaluated the patients’ medical history, radiological findings, tumor burden, and nuclear imaging studies in order to decide the best therapeutic option/target. The medical history was reviewed to classify in which treatment line the radionuclide therapy was administered. Phenotypic nuclear medicine and conventional imaging studies assessed the tumor burden.

The diagnostic phenotypic studies included in this report and performed at least 2 months prior to radionuclide treatment were as follows: [^123^I]MIBG SPECT/CT, [^111^In]In-Pentetreotide/[^99m^Tc]Tc-HYNIC-TOC SPECT/CT (on the whole SSTR SPECT/CT), [^68^Ga]Ga-DOTA-TOC PET/CT or PET/MRI (^68^Ga-DOTA-SSA), [^18^F]fludeoxyglucose PET/CT ([^18^F]FDG) and l-6-[^18^F]fluorodopa PET/CT ([^18^F]DOPA). We reviewed whether nuclear medicine images assessed the possibility to treat with only one or both therapies, and their target phenotypic pattern (transporters/receptors intensity uptake, heterogeneity, and extension). The target and purpose of the scans are summarized in **Table 1**.

**Table 1:** Target and purpose of the nuclear medicine scans used in this report.

Target	Norepinephrine transporter	Somatostatin receptor	Glucose transporter	L amino acid transporter
Functional imaging	[^123^I]MIBG SPECT/CT	[^111^In]In-Pentetreotide/[^99m^Tc]Tc-HYNIC-TOC SPECT/CT, [^68^Ga]Ga-DOTA-TOC PET/CT, [^68^Ga]Ga-DOTA-TOC PET/MR	[^18^F]fludeoxyglucose PET/CT	l-6-[^18^F] Fluorodopa PET/CT
Purpose	Diagnostic and [^131^I]MIBG therapy planning	Diagnostic and [^177^Lu] Lu-DOTA-TATE therapy planning	Diagnostic, prognostic, and metabolic.	Diagnostic and metabolic. Similarity with [^123-^I]MIBG uptake
			Allows to rule out radionuclide therapy	

According to the radiotracers’ uptake and its intensity, the patients were referred to PRRT, [^131^I]MIBG, or other non-radiolabeled systemic treatments with 4 possible scenarios:

1. Patients with obvious higher SSTR expression in ^68^Ga-DOTA-SSA or SSTR SPECT/CT were referred to treatment with PRRT.
2. Patients with similar/higher [^123^I]MIBG uptake than SSTR studies were referred to [^131^I]MIBG.
3. Heterogeneous or unclear better uptake pattern between SSTR images and [^123^I]MIBG. In this scenario, the multidisciplinary committee decided the best approach depending on the phenotypic images, the patient’s profile, and tumor features.
4. The cases with increased [^18^F]FDG uptake compared to the rest of radiotracers (especially lesions with glucose uptake and without SSTR or NET expression) were referred to other systemic treatments and were not included in this observational study.

We reviewed the functional imaging scans findings, prior systemic treatments, the genetic profile, and overall, clinical, hormonal, and radiological responses (the last one according to response evaluation criteria in solid tumors (RECIST) (7). PFS from the date of first radionuclide therapy until the last control or progression, DCR (patients with a response and disease stability), and follow-up until death or last available control were calculated. Contingency and multiparametric tests were performed on the variables under study. The contingency coefficient between PFS, therapeutic modality, previous treatments, and tumor location was evaluated, and statistical significances between them were assessed by the Mann–Whitney U test in those variables in which the samples were not dependent. The Kaplan–Meier survival plots were obtained (IBM SPSS Statistics 23).

### Peptide Receptor Radionuclide Therapy

This group involved 9 patients (5 women) with a mean age of 45.8 years (20–72 years). Inclusion criteria were a tumor uptake higher than liver reference activity (Krenning score 3–4) in SSTR molecular imaging studies, blood test values according to international guidelines, life expectancy over 6 months, and Eastern Cooperative Oncology Group (ECOG) scale <2. No patient had relative or absolute contraindications prior to therapy (8).

Possible drug interferences were reviewed to avoid medication interactions. Long-half-life SSAs were withdrawn for 4–6 weeks before therapy and short-half-life formulations at least 24 h before treatment. Premedication with alpha- and beta-adrenergic drugs was considered in those patients with increased catecholaminergic symptoms.

atients were admitted to the metabolic therapy unit for 24 h. An ondansetron premedication was administered firstly, followed by an amino acid infusion (2.5% lysine and 2.5% arginine) in 1 L of water, followed by the [^177^Lu]Lu-DOTA-TATE radiopharmaceutical infusion over 30 min. The amino acid infusion was extended for 3 h after [^177^Lu]Lu-DOTA-TATE. Standard administered activity was approximately 8.01 GBq per cycle (7.4–8.4) (SD 0.23) at a mean of 73.8 days’ intervals (50–246 days) (SD 45.8).

Complete blood tests were performed 2, 4, and 6 weeks after each cycle and 6 months after therapy. Toxicities were described according to Common Terminology Criteria for Adverse Events version 5.0. Clinical follow-up assessed the response parameters.

In patients without disease progression, SSTR molecular images were performed 3 months, 6 months, and annually after therapy. [^18^F]FDG was performed additionally during follow-up if needed. The tumoral lesions were morphologically characterized according to RECIST with CT or MRI.

### ^131^I-Metaiodobenzylguanidine

This arm included 8 patients (3 women) with a mean age of 45.5 years (5–77 years), displaying similar or higher uptake in [^123^I]MIBG than the SSTR imaging scans.

In addition to the functional imaging scans, the inclusion criteria were blood test values according to the international guidelines and life expectancy over 3 months (9).

Possible drug interferences were reviewed to avoid medication interactions, including antiarrhythmics, antihypertensives, antidepressants, or sympathomimetics, as well as calcium antagonists.

A mean dose of 6.5 GBq (3.1–8.2) (SD 40.4) was administered per cycle. The lowest dose was in a pediatric patient where it was adjusted based on weight. Previous thyroid blockade with a potassium iodide solution was performed, and alpha- and beta-adrenergic blockers were administered in those patients with risk of hypertensive crisis some weeks before therapy.

Patients were admitted to the metabolic therapy unit for 4–5 days until the 1-m dose rate allowed discharge from the hospital according to local legislation. After antiemetic premedication, on the first day, slow intravenous [^131^I]MIBG perfusion was administered during 1–4 h. In order to minimize bladder radiation, oral and intravenous hydration was encouraged.

The first response evaluation was performed most frequently after 3–6 months, except in those patients with suspected early progressive disease. Conventional imaging assessed the RECIST criteria.

## Results

### Peptide Receptor Radionuclide Therapy Results

**Figure 3:**
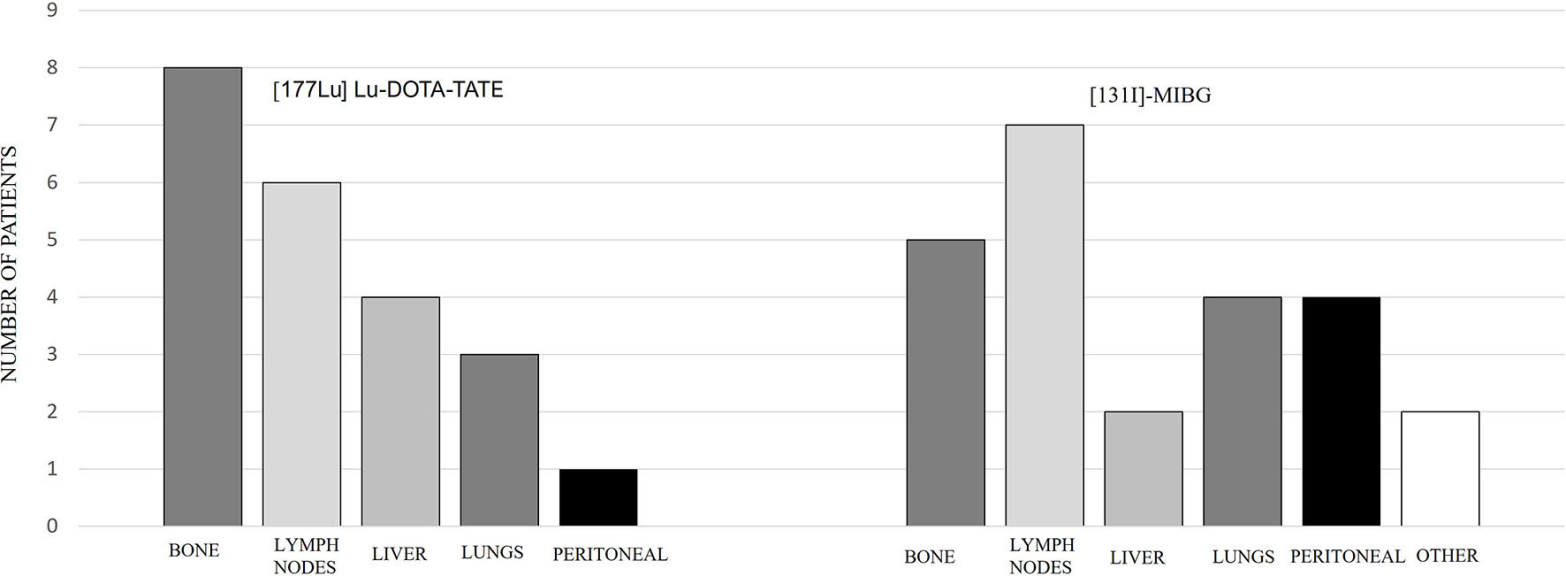
Metastatic location in [^177^Lu]Lu-DOTA-TATE and [^131^I]MIBG therapy.

Out of the 9 patients, 6 were PGLs and 3 were PHEOs. Four cases were sporadic and 5 familial, out of which 4 were related to pseudohypoxic cluster (3 SDHB and 1 SDHD) and 1 NF1 mutation related to kinase signaling cluster. One patient had a locally advanced and unresectable cervical disease, and the rest had metastatic disease. Patient characteristics are summarized in **Table 2**, and the metastatic location distribution is represented in **Figure 3**.

**Table 2:** Patient characteristics in [^177^Lu]Lu-DOTA-TATE treatment.

Patient	Age	Gender	Primary location	Mutation	Previous treatments	Previous functional image	Metastases location	Cycles	Side effects	Biochemical response	Radiological response	Clinical response	Survival	PFS	Subsequent treatments
**1**	38	F	Abdominal PGL	SDHD	SSA^a^	MIBG^d^−DOPA^e^− FDG^f^+ SRS^g^+	Lymph nodes, bone	3	No	-	PR	-	81 months	81 months	-
**2**	20	M	Left PHEO	NF1	No	MIBG− FDG+ SRS++	Bone, lung	3	GI	CR^i^	PR	SD asymptomatic	80 months	80 months	–
**3**	46	F	Abdominal PGL	Sporadic	Sunitinib, SSA, CVD^b^	MIBG+, FDG+, SRS++	Liver, lymph nodes, bone	1	Hematologic grade 3 toxicity (adverse event)	PR^j^	SD^k^	PD	5 months, exitus	0 months	No
**4**	50	F	Abdominal PGL	SDHB	SSA, TMZ^c^, vertebral cementation.	FDG+, SRS+, gallium^h^+	Bone	1	No	–	SD	SD	4 months, exitus	4 months	No
**5**	62	M	Cervical PGL	SDHB	SSA	SRS+	No (unresectable)	4	Acute cholecystitis after 2° cycle	PR	SD	SD asymptomatic	42 months	42 months	No
**6**	51	F	Left PHEO	Sporadic	SSA, bone RT.	FDG+, MIBG+/−, SRS+	Lymph nodes, bone, lung	4	Hematologic grade 1 toxicity	CR	Pulmonary CR. SD in lymph nodes and bone	SD oligosymptomatic	21 months	21 months	No
**7**	28	M	Abdominal PGL	SDHB	CVD, vertebral cementation, SSA, pelvic RT, TMZ, ^131^I-MIBG.	SRS+ FDG++, MIBG+, Gallium++	Lymph nodes, bone, lung	4	No	-	PR 10 months	SD 10 months	12 months, exitus	10 months	No
											Afterwards PD^l^	Afterwards PD			
**8**	72	M	Abdominal PGL	Sporadic	Bone RT, liver metastasectomy, SSA.	SRS+, MIBG+/−, Gallium++, FDG++	Liver, lymph nodes, bone	4	Hematologic grade 1 toxicity	–	SD	SD	17 months	17 months	No
**9**	45	F	Right PHEO	Sporadic	SSA, abdominal implant resection, ^131^I-MIBG.	SRS+ MIBG+Gallium++	Liver, lymph nodes, bone, pelvic implants	4	Hematologic grade 1 toxicity, GI	-	SD	SD	6 months	6 months	No

a Somatostatin analogs.

b Cyclophosphamide, vincristine, and dacarbazine chemotherapy.

c Temozolamide.

d [^123^I]MIBG scintigraphy.

e l-6-[^18^F]fluorodopa PET/CT.

f [^18^F]Fludeoxyglucose PET/CT.

g Somatostatin receptor scintigraphy.

h [^68^Ga]Ga-DOTA-TOC PET/CT.

i Complete response.

j Partial response.

k Stable disease.

l Progressive disease.

++, very intense uptake; +, intense uptake; +/−, low or heterogeneous uptake; −, very low or absent uptake.

In 7 cases, functional imaging studies revealed higher uptake in SSTR SPECT/CT or ^68^Ga-DOTA-SSA compared to [^123^I]MIBG (with appropriate uptake of both targets in 3 patients), and in 2 cases, only SSTR SPECT/CT was performed. In 7 patients, we acquired an additional [^18^F]FDG scan that showed always fewer metabolic lesions than those expressing SSTRs.

Only 1 patient was treated with PRRT as first-line therapy, and the largest group was treated with second-line therapy, having previously received SSA and [^131^I]MIBG. Two patients showing similar uptake with both SSTR imaging scans and [^123^I]MIBG were previously treated with [^131^I]MIBG. In these cases, the multidisciplinary committee considered PRRT as the second choice radiolabeled therapy due to the presence of radiological progression to [^131^I]MIBG and sufficient expression of SSTRs.

The average cumulative administered radioactivity was 24.9 GBq (8.5–32.4), with a mean of 3 cycles (1–4 cycles). Two patients received only 1 cycle due to rapid progressive disease (PD). Three patients died with a mean time from treatment to death of 7 months (4–12 months). In those cases, PRRT was generally administered in a late therapeutic line (3rd to 5th line), two of them carried SDHB mutation, and all of them had bone involvement.

The overall PFS was 29 months (0–81 months) with a mean follow-up of 29.78 months (4–81 months). In the PGL group, the PFS was 25.6 months (22.4 months for the sympathetic and 42 months in the cervical parasympathetic case), and it was 35.6 months in the 3 patients with PHEO.

**Figure 4:**
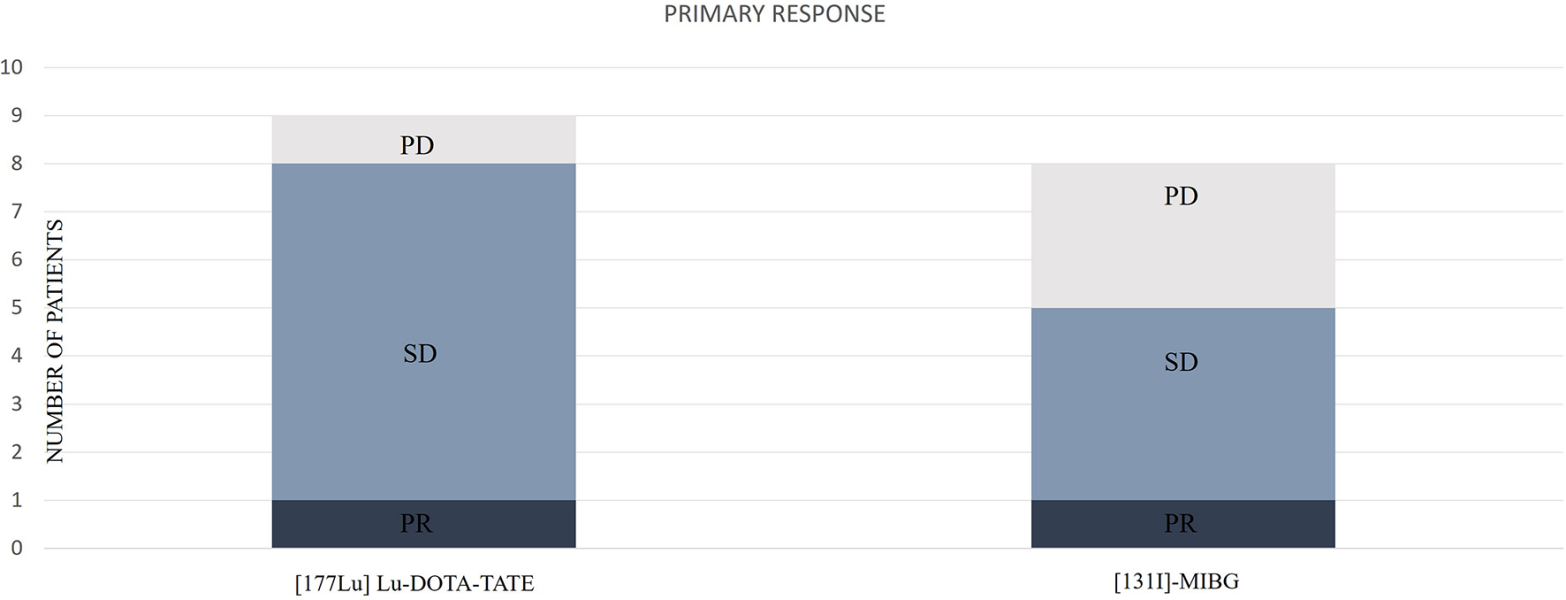
Distribution of the primary responses according to [^177^Lu]Lu-DOTA-TATE or [^131^I]MIBG. PD, progressive disease; SD, stable disease; PR, partial response.

We observed an initial DCR of 88.8%. From patients with follow-up, initial biochemical DC was observed in 4 cases (100%), radiological DC in 9 (100%), and clinical DC in 7 (87.5%). The distribution of the primary responses is represented in **Figure 4**.

Hematological grade 1 toxicity and gastrointestinal symptoms were the most common side effects (33.3% and 22.2%, respectively). The most frequent hematological toxicities observed were mild anemia and thrombocytopenia, which eased spontaneously and did not require the treatment dose interval to be lengthened. Within gastrointestinal symptoms, the most common were mild nausea or vomiting. Patient number 3 had a grade 3 hematological toxicity (severe thrombo and leukopenia), which did not seem directly related to PRRT but was probably induced by prior cyclophosphamide, vincristine, and dacarbazine chemotherapy (CVD). Patient number 5 presented cholecystitis after the second cycle in the context of multiple liver metastases and previous SSA treatment.

### ^131^I-Metaiodobenzylguanidine Results

Eight patients were treated in this group, 3 were extra-adrenal abdominal PGLs and 5 PHEOs, 4 cases were sporadic, 2 carried an SDHB mutation, and 2 lacked genetic testing. Patient features are summarized in **Table 3**.

**Table 3:** Patient characteristics in [^131^I]MIBG.

Patient	Age	Gender	Primary location	Mutation	Previous treatments	Previous functional image	Metastases location	Cycles	Side effects	Biochemical response	Radiological response	Clinical response	Survival	PFS	Subsequent treatments
**1**	5	M	Abdominal PGL	–	^131^I-MIBG 2 cycles in another center	MIBG+, FDG+	Lymph nodes, bone	1	No	CR	PR	CR	77 months	77 months	No
**2**	36	M	Pelvic PGL	SDHB	Non specified chemotherapy. Bone thermoablation	MIBG++, FDG++	Lung, lymph nodes, bone, liver, abdominal implants	2	No	–	–	SD 6 months, Loss of clinical follow-up	50 months	6 months	–
**3**	65	M	Left PHEO	Sporadic	No	MIBG+, FDG+	Lung, lymph nodes	1	No	SD	SD 20 months, afterwards PD	SD 20 months, afterwards PD	49 months	20	CVD
**4**	77	F	Left PHEO	–	CVD	SRS+/−, MIBG++	Lung, lymph nodes, abdominal implants	1	No	PD	PD	PD	3 months, exitus	0	–
**5**	27	M	Abdominal PGL	SDHB	CVD, bone cementation, SSA, pelvic RT, TMZ	SRS+, MIBG+	Bone, lymph nodes	1	Hypertensive crisis	PD	SD 3 months, afterwards PD	SD 6 months, afterwards PD	32 months, exitus	0	PRRT 4 cycles, CVD
**6**	43	F	Right PHEO	Sporadic	SSA, abdominal implant resection	SRS+, MIBG++	Lymph nodes, bone, abdominal implants	1	Hypertensive crisis with Valsalva maneuver	PR 21 months, afterwards PD	SD 21 months, afterwards PD	SD 21 months, afterwards PD	31 months	21 months	PRRT 4 cycles
**7**	63	F	Right PHEO	Sporadic	Liver metastasectomy nephrectomy, RT	SRS−, MIBG+	Lymph nodes, bone, liver, kidney	1	No	PR	SD	SD	24 months	24 months	No
**8**	48	M	Left PHEO	Sporadic	CVD, CAPTEM^a^, SSA	Gallium(PET/MRI)+, MIBG+ DOPA++, FDG++	Lung, Lymph nodes, abdominal implants, spleen	1	Hematologic grade 2 toxicity, GI	–	–	PD	3 months	0 months	No

a Capecitabine and temozolomide.

++, very intense uptake; +, intense uptake; +/−, low or heterogeneous uptake; −, very low or absent uptake.

Three patients had previous functional imaging with [^123^I]MIBG and [^18^F]FDG, which were concordant. In 4 patients, SSTR SPECT/CT and [^123^I]MIBG were performed with similar-higher avidity for [^123^I]MIBG. One patient had undergone an [^18^F]DOPA, an [^18^F]FDG, and, in the context of a research project, a PET/CT-PET/MRI protocol with ^68^Ga-DOTA-SSA. In this last patient, while the first two scans were consistent, in ^68^Ga-DOTA-SSA, the lesions showed less uptake intensity than the liver. The PET/MRI helped us characterize cystic-necrotic lesions, especially in a doubtful lesion on CT located in the splenic capsule.

In 3 patients, [^131^I]MIBG was the first choice systemic treatment; in another 3, it was the second option; and finally in 2 cases, it was the fourth therapeutic line.

The average cumulative administered radioactivity was 7.39 GBq (3.1–12.33). Seven patients received 1 cycle, and in one patient with wide disease extension, 2 cycles were administered at a 2-month interval.

Overall PFS was 18.5 months (0–25) with a 33.6-month follow-up (3–77 months). In the PGL group, the PFS was 27.6 months, and in the PHEO group, it was 13 months.

Three patients died, with a mean time from treatment to death of 18.33 months (1–42 months). One received CVD previously, and the other two patients were treated in a fourth-line scheme.

We observed an initial DCR of 62.5%. From patients with follow-up, initial biochemical DC was observed in 4 cases (66.6%), radiological DC in 5 (83.3%), and clinical DC in 6 (75%).

One case had a severe hypertensive event that required specific antihypertensive intravenous medication, and another one experienced high blood pressure associated with the Valsalva maneuver. We reported one gastrointestinal side effect and a grade 2 hematological toxicity adverse event with anemia and thrombocytopenia.

### Overall Results

**Figure 5:**
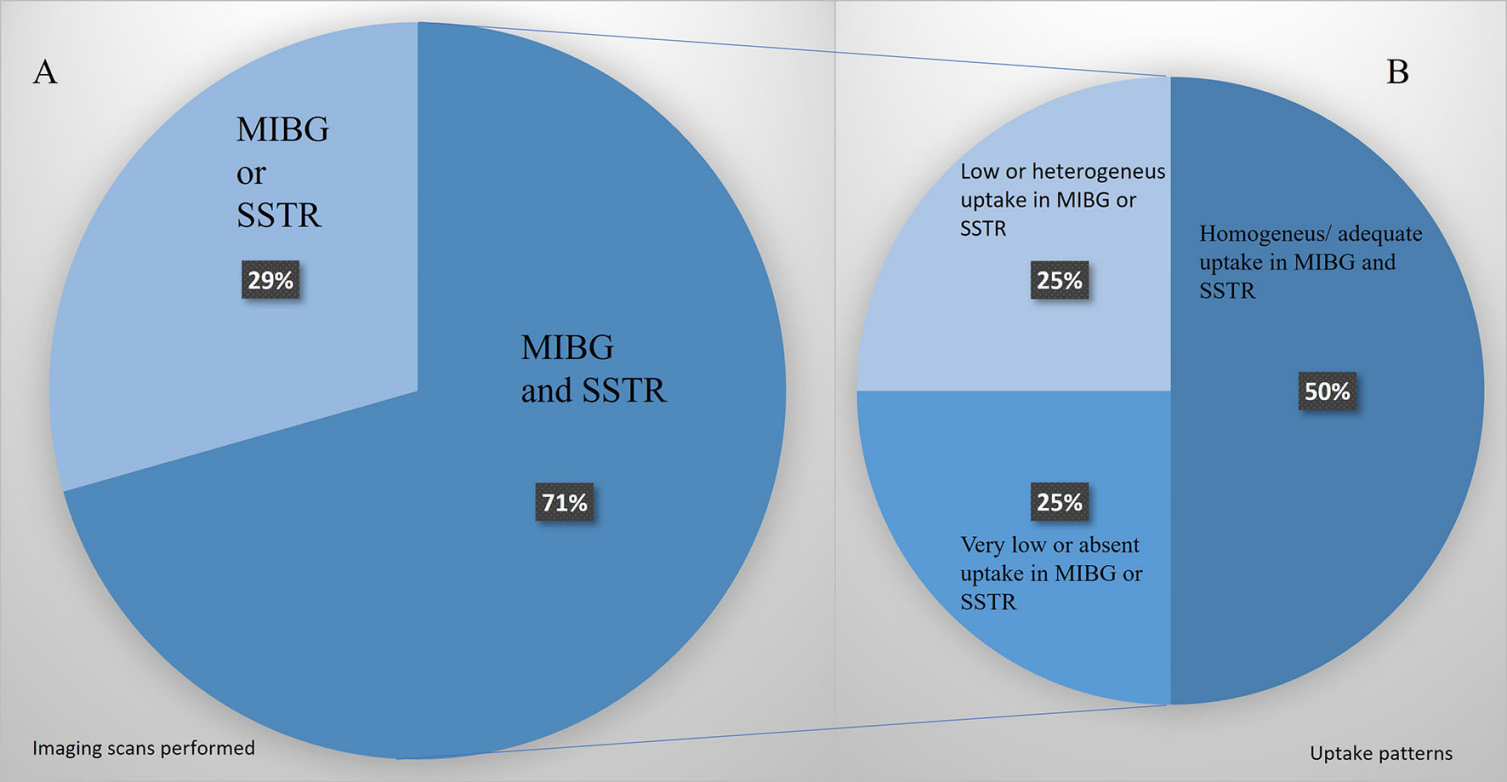
**(A)** Patients’ percentage assessed only by [^123^I]MIBG or SSTR images vs. those assessed by the two image modalities. **(B)** Uptake pattern distribution in those patients studied with both image modalities.

Not all the patients were candidates for both therapies. The phenotypic images, patient’s profile, and tumor features selected 9 patients for PRRT and 8 for MIBG. In 5 patients, only an [^123^I]MIBG or SSTR image was performed, and if positive, the patients were treated with the corresponding modality. In 12 patients, [^123^I]MIBG and SSTR images were performed, and in 6 of them (50%), there was an appropriate uptake of both radiotracers (**Figure 5**). In these cases, a multidisciplinary committee decided to use that therapy with higher target uptake and/or more tailored to the clinical context as referred above. In the other 50%, we found significant heterogeneity, low or absent uptake that excluded one of the therapeutic modalities.

**Figure 6:**
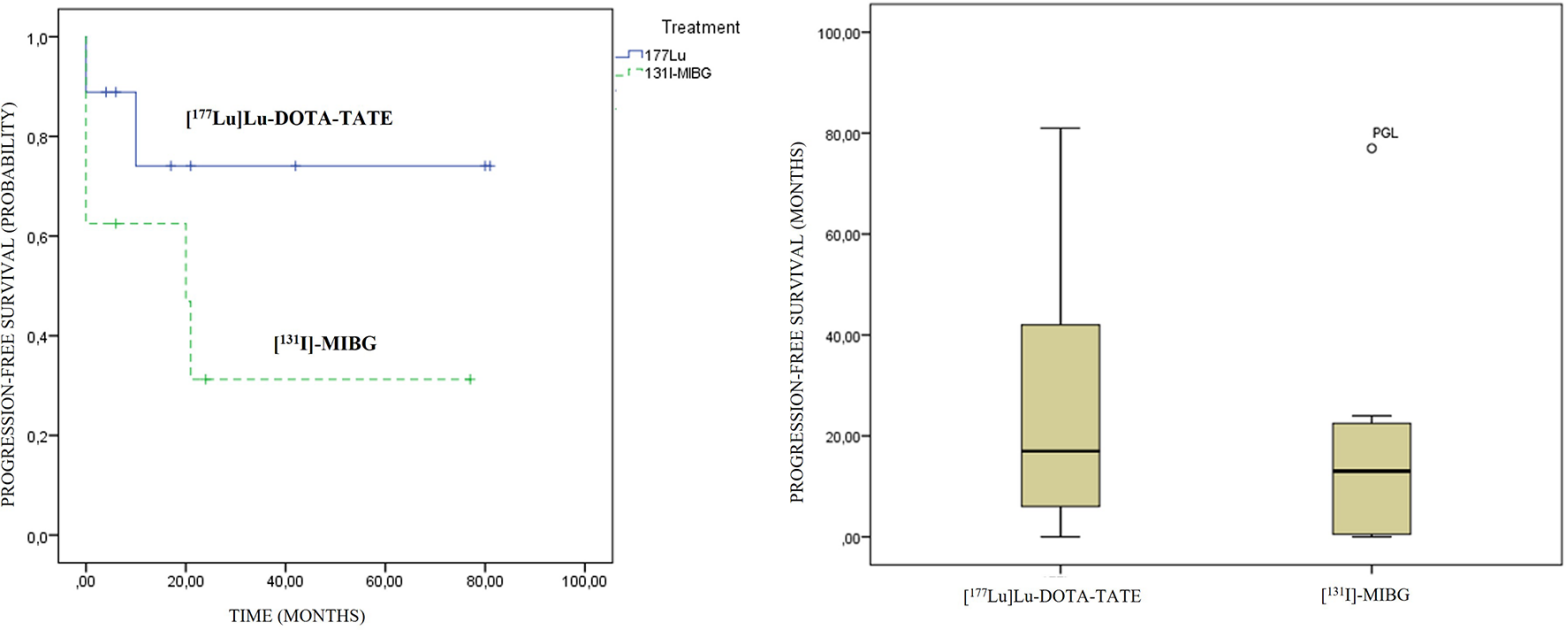
[^177^Lu]Lu-DOTA-TATE and [^131^I]MIBG progression-free survival graphics. On the left side, Kaplan–Meier curves; and on the right side, box plots are presented.

An association between the variables’ previous treatments and PFS was observed (contingency coefficient of 0.879), with a trend to lower survival as the treatment was administered in a later line. No statistically significant differences were observed in PFS regarding primary location and therapeutic modality. A longer PFS in the PRRT modality was observed compared to [^131^I]MIBG, 29 vs. 18.5 months, respectively, although they did not show statistically significant differences (p: 0.481) (**Figure 6**).

**Figure 7:**
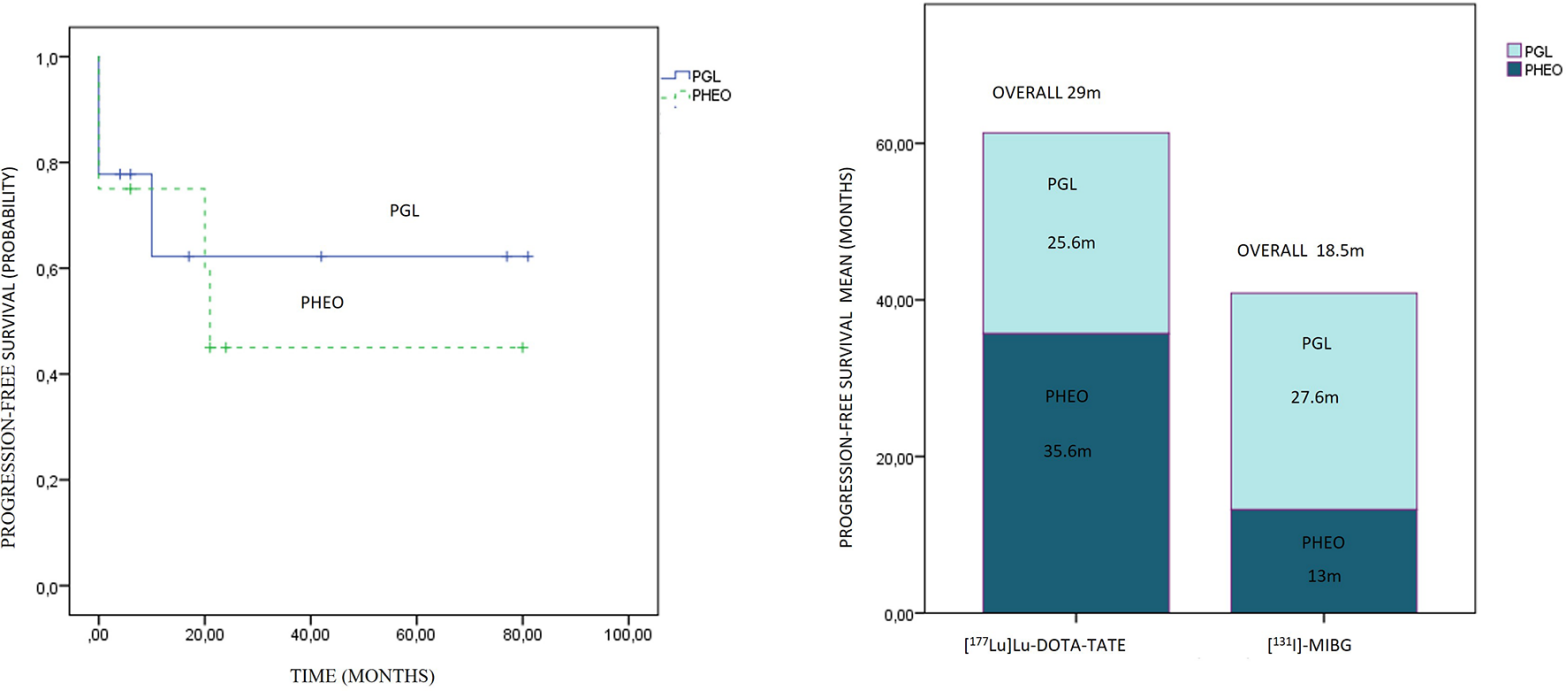
On the left side, progression-free survival Kaplan–Meier plot related to paragangliomas and pheochromocytomas is presented. On the right side, the progression-free survival distribution according to treatment and primary location is presented.

Comparing the subgroups, the overall PFS was 26.3 months for PGLs and 21.5 months for PHEOs, with a follow-up of 35.5 and 26.9 months, respectively, with no statistical significance (p: 0.743) (**Figure 7**). The overall PFS with PRRT vs. [^131^I]MIBG was respectively 25.6 vs. 27.6 months for PGLs, and 35.6 vs. 13 months for PHEOs. The most frequent location of metastatic lesions was similar in the two therapies, predominantly in bone and lymph nodes.

## Discussion

Reports related to radionuclide therapy with PRRT and [^131^I]MIBG are usually heterogeneous case series, in procedure and data collection, with few patients and some of them not differentiating PHEO and PGL subgroups, without any clear data on PFS nor follow-up. There are often differences in [^131^I]MIBG treatment, particularly in patient selection and in the number of doses and cycles to be administered. The lack of consensus and a clear therapeutic guide underpin this study.

The phenotypic imaging scans used to display the targets for radiolabeled therapy have been recently improved with novel functional imaging equipment and radiopharmaceuticals, modifying the image explorations performed over our study. The recent availability of PET/CT in the nuclear medicine departments, with higher spatial resolution than SPECT/CT or the trend of PET/MRI from the research area to the clinical application, allow to characterize higher uptakes in smaller lesions that previously went underestimated or unnoticed (10). In recent literature, Crona et al. (2017) and Nölting et al. (2019) recommend to compare ^68^Ga-DOTA-SSA with [^123^I]MIBG in order to decide the best therapeutic option (11). However, in fact, these scan modalities are not fully comparable from a technical point of view and thus could lead to an underestimation of the NET expression, rejecting possible candidates to [^131^I]MIBG. Although in the guidelines the proven tumor uptake in [^123^I]MIBG scintigraphy is essential to plan [^131^I]MIBG treatment, some reports lay out a high concordance degree between ^18^F-DOPA and posttreatment distribution scintigraphy with [^131^I]MIBG, as it happened in our patient number 8 in MIBG table (12–14). [^124^I]MIBG radiotracer for PET scan is also an encouraging future option to assess the possibility of both treatments under the same conditions.

To our knowledge, this is the first published study comparing the selection and the response to treatment with full series of [^177^Lu]Lu-DOTA-TATE and [^131^I]MIBG in PGGLs.

PRRT reported series in literature are quite homogeneous with cumulative radioactivities of between 22 and 29.6 GBq and similar PFS results to ours. Compared with studies with similar follow-up, Yadav et al. (2019) treated 25 patients with a cumulative dose of 22.8 GBq concomitantly with capecitabine, achieving a 32-month PFS with a follow-up of 30 months (15). Zandee et al. (2019) with a longer follow-up (52.5 months) obtained a 30-month PFS in 10 patients with parasympathetic PGL; 13-month PFS in the sympathetic cases; and 8, 10, and 14 months in the 3 PHEO patients (16).

In our PRRT series, there was a longer PFS in PHEOs than in PGLs. This was probably due to 3 exitus in PGLs with advanced disease and high comorbidity, receiving therapy in a range from the third to fifth line.

On the other hand, [^131^I]MIBG therapy reports are quite heterogeneous in cumulative dose (between 3.7 and 39.4 GBq) and the number of cycles (1–10 cycles). It is unclear if a higher dose results in a higher tumor response or dosimetry. Castellani et al. (2010) compared two groups using intermediate vs. low cumulative dose (39.4 vs. 24.1 GBq) and observed a small variation in PFS with 30 vs. 24.92 months, respectively. They concluded that the most important difference between the groups was the shortened time to achieve a significant response (17).

Two of the studies with cumulative doses closer to ours provide a similar PFS. Shilkrut et al. (2010) with 1–4 cycles and 11.6 GBq observed a 17.5-month PFS, and Rachh et al. (2011) with 1–5 cycles and 11.8 GBq observed a PFS of 29 months (18, 19). Similar results have also been observed by Fishbein et al. (2012) who combined 1–3 cycles of [^131^I]MIBG and radiotherapy with an 11.26-month PFS and by Gedik et al. (2008) with 1–10 cycles of 22.2 GBq (three times more than our series) with a 28.5-month PFS. In this context, we administered a lower cumulative dose than most of those described in the literature, without any significant differences in the PFS results. Comparing by subgroups, our [^131^I]MIBG series results agree on the recent reviews that provide a more time-sustained response to treatment for PGLs than for PHEOS (27.6 vs. 13 months) (20, 21). We also observed fewer adverse events, with only one grade 2 thrombocytopenia (12.5%), probably related to the lower dose administered (22).

**Figure 8:**
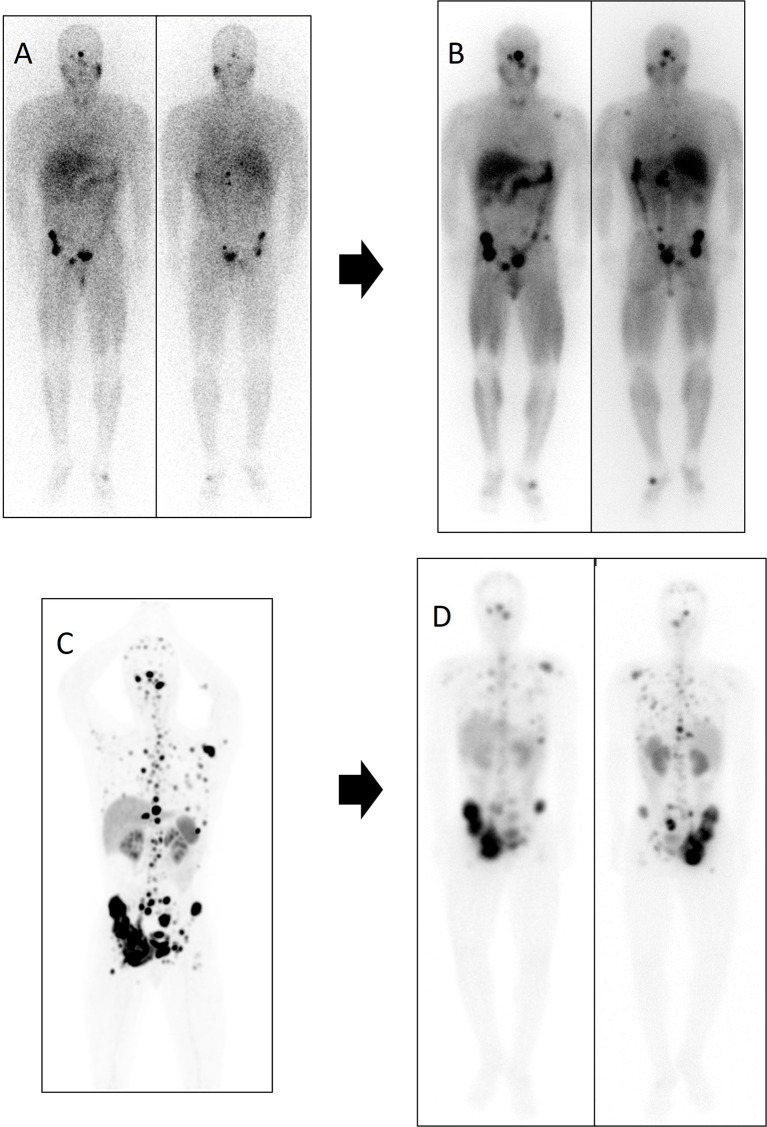
Patient number 5 in the [^131^I]MIBG table was treated first with [^131^I]MIBG and subsequently with PRRT. After 6 months of disease stability with [^131^I]MIBG, lung, lymph node, and the bone disease progressed. Subsequently, the patient received treatment with [^177^Lu]Lu-DOTA-TATE, reaching disease stability of 10 months. Diagnostic functional imaging studies and post-therapy distribution scans of both therapies are shown. **(A)** Diagnostic scan with [^123^I]MIBG in anterior and posterior planar projection. **(B)** Distribution scan after treatment with 7.4 GBq of [^131^I]MIBG in anterior and posterior planar projection. **(C)** ^68^Ga-DOTA-SSA PET/CT maximum intensity projection (MIP). **(D)** Distribution scan after treatment with 8.1 GBq of [^177^Lu]Lu-DOTA-TATE in anterior and posterior planar projection.

In our series, sequential treatment with both radionuclides was achieved in two patients (5 and 6 of [^131^I]MIBG series) (**Figure 8**). Due to a PD after [^131^I]MIBG therapy, both received 4 cycles of PRRT. The first patient died without hematological toxicity with an overall clinical PFS of 16 months, and the second patient remains in stable disease, achieving 37-month PFS and only grade 1 hematological toxicity. These results suggest that a sequential radionuclide therapy with both modalities could be a future alternative especially in those patients with aggressive disease. Similar findings were described by Nastos et al. (2017) in two patients, one treated with [^90^Y]Y-DOTA-TOC and the other with [^177^Lu]Lu-DOTA-TATE, reporting grades 4 and 2 of hematological toxicity, respectively. Although our cumulative dose was in the range of those described by Nastos et al., probably in our case, the hematological toxicity was lower due to a longer delay of 10 and 12 months between both sequential treatments (23). Recently, Bushnell et al. (2021) reported a phase 1 clinical trial combining treatment with [^90^Y]Y-DOTA-TOC and [^131^I]MIBG in 3 patients with neuroendocrine tumors. This association allowed them to increase the dose of radiation to the tumor with an adequate margin of safety (24).

We observed a trend towards longer PFS (29 months) and DCR (88%) with PRRT and especially in PHEOs. Given that in our series approximately half of the cases overexpressed both targets and therefore were potential candidates to both therapeutic modalities, a better approach to the treatment responses could guide the selection of the most appropriate and precise sequencing.

The current survival rates may be modified in the near future with the approval of the high-specific-activity (HSA) [^131^I]MIBG by the Food and Drug Administration (FDA) in 2018. In the LSA [^131^I]MIBG used to date, more than 99% of MIBG molecules are not labeled with ^131^I (“cold” MIBG). The “cold” MIBG competes with the radiolabeled molecule in the reuptake process by NETs and increases the side effects. In the HSA, the labeling process is 100–200 times more efficient, reducing side effects and competitiveness with the “cold” MIBG. [^131^I]MIBG (HSA) has shown in a prospective study in 68 patients with PHEOs and PGLs a sustained reduction of hypertensive medication, objective partial tumor responses of 23%, and stable disease in 69% (92% DCR). With this emerging alternative, a real intermodality agreement, in terms of spatial resolution and intensity uptake, will be required in the pretherapeutic scans (25).

Among the study limitations, the first one is that this is a retrospective case review. The PRRT and [^131^I]MIBG are not entirely comparable. Depending on the molecular tumor features and according to our results, at least 50% of the cases will not be candidates for both therapies. The series is small due to the low disease prevalence and does not allow to yield statistically significant differences on multiparametric tests and the survival rates in potential candidates to both treatments. The functional imaging studies are heterogeneous and have changed over time. The lack of a longer follow-up did not allow to reach the overall survival goal and to rule out late toxicities. Due to the slow progression and the disease stability periods in some patients, response to treatment could have been overestimated.

## Conclusion

In the context of an infrequent disease with scarce therapeutic options, both [^177^Lu]Lu-DOTA-TATE and [^131^I]MIBG are safe, with a promising initial DCR and a trend towards the best results in PRRT series and PHEOs. In these patients, choosing the most suitable radiolabeled treatment modality is a clinical dilemma that requires a multidisciplinary committee, and it is supported by the development of new phenotypic images. Altogether, not only the functional imaging uptake should be evaluated. Comorbidities, patient profile, tumor features, and better response rates should also be kept in mind. The patients with the worst prognosis were those treated in later lines and with greater tumor burden. Both therapies should be studied in earlier scheme lines and with larger prospective series in order to review therapeutic selection and sequence. Although the results present limitations and the sample size and the heterogeneity of the study population do not allow to define which is the best imaging pattern to predict the response to treatment, this should be the first step to carry out a prospective study, to establish which patients would benefit from one of the two treatments and, if both can be administered, the most suitable sequence.

## Data Availability Statement

The original contributions presented in the study are included in the article/supplementary material. Further inquiries can be directed to the corresponding author.

## Ethics Statement

The studies involving human participants were reviewed and approved by Hospital Universitario y Politécnico La Fe, FPNT-CEIB-04 (B). N° de registro: 2020-432-1. The patients/participants provided their written informed consent to participate in this study.

## Author Contributions

SP-W is the main author. MO-G, PB-A, and JM-T collaborated in the writing and revision of the article. All authors listed have made a substantial, direct, and intellectual contribution to the work and approved it for publication.

## Funding

This study was funded by the University Hospital and Polytechnic Research Institute of Valencia, Spain.

## Conflict of Interest

The authors declare that the research was conducted in the absence of any commercial or financial relationships that could be construed as a potential conflict of interest.

## Publisher’s Note

All claims expressed in this article are solely those of the authors and do not necessarily represent those of their affiliated organizations, or those of the publisher, the editors and the reviewers. Any product that may be evaluated in this article, or claim that may be made by its manufacturer, is not guaranteed or endorsed by the publisher.
